# A New Method of Treating Disease by Controling the Circulation of the Blood in Different Parts of the Body

**Published:** 1864-09

**Authors:** John Chapman


### SELECTED.

### A NEW METHOD OF TREATING DISEASE BY
### CONTROLING THE CIRCULATION OF THE BLOOD IN DIFFERENT PARTS OF THE BODY.

By JOHN CHAPMAN, M. D., M. R. C. P.

It has long been known that the sympathetic nerve, called by Bichat, the nervous system of organic life, presides over those processes by which the body is developed and sustained. It stimulates and controls the action of the heart, alimentary canal, genito-urinary organs, and all those processes of growth, repair, and removal of *effete* materials on which the continuous vitality and health of the animal organism depend. During recent years, important additions to our knowledge of the functions of the sympathetic nerve have been made, chiefly by Prof. Claude Bernard and Dr. Brown-Séquard, especially with reference to its power of controlling the action of blood-vessels, or what have been termed its vaso-motor functions. But as the sympathetic and cerebro-spinal systems are intimately related, and, indeed, in some parts, inextricably and indistinguishably blended, both in structure and function, the nervous influence, whether healthy or not, which is exerted over the several organs of the body, is two-fold ; hence, when that influence becomes abnormal, either in kind or degree, the most potent method of restoring it to its healthy condition, would be by a dual action at once on the sympathetic and cerebro-spinal nervous systems. The physician who acquires the power of directly controlling these great controllers of the organic functions would immediately obtain the mastery over a large number of diseases. I scarcely dare write the words, " I have done this"—so momentous are they if true ; and yet I believe I have.

I have discovered that a controlling power over the circulation of blood in the brain, in the spinal cord, in the ganglia of the sympathetic nervous system, and, through the agency of these nervous centres, also in every other organ of the body, can be exercised by means of cold and heat applied to different parts of the back. In this manner the reflex excitability, or excito-motor power of the spinal cord, and the contractile force of the arteries in all parts of the body can be immediately modified.

In order to lessen the excito-motor of the spinal cord only, I apply ice, in an India-rubber bag about two inches wide, along that part of the spinal column containing the part of the cord on which I wish to act. On the same principle, the vitality of the spinal cord may be increased by applying hot water and ice alternately, each in an India-rubber bag, if very energetic action be required; if less vigorous action be necessary, I apply ice, or iced water only, using it several times a day, for a short time on each occasion, with a long interval between each application.

If it be desirable to increase the circulation in any given part of the body, this I have found myself able to effect by exerting a soothing, sedative, depressing, or paralyzing influence (according to the amount of power required) over those ganglia of the sympathetic which send vaso-motor nerves to the part intended to be acted on. This influence may be exerted by applying ice to the central part of the back, over a width of from four to four and a half inches, and extending longitudinally over the particular segments of the sympathetic and of the spinal cord on which it is desired to act.

For example, intending to direct a fuller and more equable flow of blood to the brain, I apply ice to the back of the neck and between the scapulæ; increased circulation and warmth of the upper extremities are induced in the same way; the thoracic and abdominal viscera can be influenced in like manner by application to the dorsal and lumbar regions; while the legs and the coldest feet ever felt can have their circulation so increased that they become thoroughly warm, by an ice-bag applied to the lower part of the back.

The bags I use are of different lengths; of the width already named for adults, and of lesser widths, of course, for children. I have had them made both of India-rubber, and of linen with a surface of India-rubber upon it: the former are the best. The width of the bags is equal throughout, except at the opening, which is narrowed to facilitate tying, and elastic to admit easily the lumps of ice. When the bag is full, I divide it, if a long one, into three segments; this can be done by constricting it forcibly with a string; the ice of the upper part is thus prevented from descending, as the melting goes on, into the lower part of the bag. I am preparing a bag on a new principle, which will be a great improvement on those I now use; but as it is not yet complete, I abstain from describing it here. I sustain the bag in the position intended by means of ribbon or tape passed through loops at the back of it, then over the shoulders, and round the body.

Theoretically, I feel assured that by the methods I have described physicians will be able to control the great majority of diseases; experimentally, I have received numerous and wonderful proofs that this assurance is well founded. By thus acting, by means of cold or heat, or both alternately or combined, on the spinal cord and ganglia of the sympathetic, I have succeeded in completely arresting the fits of many epileptics, and in curing the following maladies:—Paralysis, long continued and extreme headaches, prolonged giddiness, extreme somnolence, a feeling of want of firmness in standing and of security in walking, habitual hallucinations, loss of memory, weakness and dimness of sight, ocular spectra, inequality of the pupils, lateral anæsthesia, incontrollable spasmodic opening and shutting of the mouth, cramps of the limbs (in two cases of the hands, incapacitating the patients to continue their work), numbness of the fingers, incapacitating the patient to pick up small objects, or to use a needle, paralysis of the bladder, incapacity to retain the urine more than a few minutes (two cases recovered to a surprising extent), profuse and too frequent menstruation, scanty and irregular menstruation, extreme menstrual pains, profuse leucorrhœa, with long continued bearing down of the womb, and extreme pain of the back, habitual constipation, habitual diarrhœa, general coldness of the surface of the body which has continued for many years, habitually and hitherto irremedially cold feet.

For the sake of brevity I abstain from discussing here the applicability of the method above described to the several diseases which the physician is called upon to treat; but as many cases of paralysis and a very great majority of cases of epilepsy have hitherto proved incurable, I will in respect to these diseases, and especially in respect to epilepsy, make a few observations, and give the briefest outlines of a few cases, showing the results of my method of treating the last-named disease.

To cure paralysis primarily originating in a lesion, not of the brain but of the spinal cord, it is necessary to exert a curative influence, not only over the spinal cord, but also over the sympathetic nervous system, to the extent of its distribution of its vaso-motor nerves throughout the paralyzed limb; to how slight an extent this has hitherto been possible, either by internal medicines or external applications, is too well known to need description here. Assuming the general truthfulness of the doctrines of Messrs. Kussmaul and Tenner, Schroeder van der Kolf, and of Dr. Brown-Sequard, respecting the essential nature of epilepsy, or the proximate cause of convulsive affections generally, we must become still more deeply impressed with the conviction of the necessity of influencing both the cerebro-spinal and the sympathetic nervous system, in order to exert any lasting curative power over that remarkable group of maladies. In Messrs. Kussmaul and Tenner's general summary of the results of their investigations concerning the nature and origin of epileptic convulsions, they say:—"It is probable that certain forms of epilepsy result from a spasm of the muscular coats of the cerebral arteries," and elsewhere they observed that if so, "the central point from which these (spasms) arise would consequently lie in the part from which the vaso-motory nerves take their origin, and therefore, if the result of Schiff's researches be correct, in the medulla oblongata. An excitement of this nervous centre would then be the first link in the chain of these processes, anæmia of the brain the second, and the epileptic attack the third." Dr. Brown-Sequard, in his "Researches on Epilepsy," after giving his reasons for thinking that "Epilepsy depends in a great measure on an increased reflex excitability of certain parts of the cerebro-spinal axis," proceeds to account for the successive phenomena of an epileptic attack, and, referring to one of the first of them—the paleness of the face—remarks, "We consider it a most interesting symptom, as it leads to a very probable explanation of the loss of consciousness in epilepsy. After Prof. Claude Bernard had discovered that the section of the cervical sympathetic nerve is followed by a dilatation of the blood-vessels of the face, I found that when this nerve is irritated by galvanism there is a contraction of these blood-vessels, and I explained the facts discovered by the eminent French physiologist and myself, by considering the sympathetic as the motor nerve of the blood-vessels of the face. I found, also, that the branches of the sympathetic nerve which animate the blood-vessels of the face, originate from the spinal cord with the branches of the same nerve going to the iris. When the excitation takes place in the spinal cord and the basis of the encephalon which gives rise to the fit, the nerve-fibres which go to the head are irritated, and produce a contraction of its blood-vessels. Of course this contraction expels the blood, and in consequence the face becomes pale. We think that at nearly the same time, when the origin of the branches of the sympathetic nerve going to the blood-vessels of the face receive an irritation in the beginning of a fit of epilepsy, the origin of the branches of the same and other nerves, going to the blood-vessels of the brain proper, also receive an irritation. A contraction then occurs in these blood-vessels, and particularly in the small arteries. This contraction expelling the blood, the brain loses at once its functions, just as it does in a complete syncope."

Though Prof. Schroeder van der Kolf differs somewhat from Messrs. Kussmaul and Tenner, and Dr. Brown-Sequard, in maintaining that the abnormal changes constituting the proximate cause of epilepsy, are more exclusively restricted to the medulla oblongata than is believed to be the case by those investigators, yet all these distinguished pathologists agree in recognizing the very important part performed by the vaso-motor nerves in producing an epileptic fit; and, though they differ as to the relative frequency of the cases in which the medulla oblongata is the primary seat of the attack, they also agree that it is very often the originating centre of the malady. It may, therefore, be stated that they all concur in the opinion that the proximate cause of epilepsy is two-fold, viz., an undue reflex excitability of the medulla oblongata, and an undue irritability of those branches of the sympathetic nerve which are distributed to the cerebral arteries, and which, in their abnormally excitable condition, induce spasmodic contractions of the cerebral blood-vessels, and the consequent loss of consciousness and fall, which usually usher in an epileptic fit. I agree with Dr. Brown-Sequard in believing that different segments of the spinal cord, as well as the medulla oblongata, are not infrequently the primary seat of epilepsy; indeed concerning the pathology of epilepsy, there is, so far as I know only one point in which I differ from that profound physiologist and skillful physician, under whose guidance, at the Hospital for Diseases of the Nervous System, it has been my good fortune to study. It is true that that "one point" is an important one, since, by diverging in the direction I have taken, I was led by a logical process to conceive of the method of curing epilepsy, which, when months afterwards, I was enabled to put it to the test of experiment, realized my sanguine expectations. For the sake of brevity, however, in this preliminary statement, I shall avoid discussing the point referred to. My immediate object in touching on the pathology of epilepsy at all at present, is merely to show that while, as I have said, "to cure paralysis primarily originating in a lesion, not of the brain, but of the spinal cord, it is necessary to exert a curative influence, not only over the spinal cord, but also over the sympathetic nervous system, to the extent of the distribution of its vaso-motor nerves throughout the paralyzed limb," so, in a like manner, to cure epilepsy it is necessary to exert a curative influence not only over the spinal cord, including the medulla oblongata, but also over the sympathetic nervous system to the extent of the distribution of the vaso-motor nerves throughout the encephalon.

In treating paralysis according to the method above described, my first effort is directed to the spinal cord, which I endeavor to restore to a healthy condition by increasing or diminishing the circulation of blood in it. I effect either of these results by directly modifying its temperature. Moreover, as fibres from the ganglia of the sympathetic are distributed to the sheaths and blood-vessels of the spinal cord, it can be influenced by cold and heat, not only directly, but indirectly, by acting on those ganglia. The restorative power which I have been able to exert in this manner is truly surprising, and I believe quite unparalleled by any influence ever exerted by medicine.

If the paralyzed limb be cold, my next object is to increase the circulation in it; this I do, as already said, by lessening the vaso-motor power of those ganglia of the sympathetic which preside over the blood-vessels of the limb in question. In this manner I find that the circulation in it can be so increased as to make it even very unpleasantly hot.

The health of the spinal cord having been improved, and the circulation and consequent nourishment of the paralyzed limb having been adequately increased, I then, and not until then, apply galvanism to the paralyzed muscle, if this aid seems needful. When thus applied, after the cord and limb have been acted on as described, the affected muscles prove far more rapidly responsive to the galvanic stimulus than paralyzed muscles usually are, and recover their natural size and strength with proportionate rapidity. But in fact, such is the health-giving influence of the processes I have described, that the limb will generally recover its healthy condition without the use of galvanism at all.

The treatment thus described has reference to those forms of paralysis originating in a lesion of the spinal cord; but I have found myself able to exert a curative influence scarcely less potent even when the paralyzing lesion is within the skull and certainly far more so than can be exerted by any internal remedy.

I could support these statements by several illustrative cases, but shall only venture to extend this paper by giving a few facts in evidence of the power over epilepsy, which my method of treatment places within the reach of the physician.

In order to cure epilepsy, care must of course be taken in the first place that all sources of eccentric irritation be removed, assured of this, as far as possible, I direct all my efforts to accomplish two objects—first, to lessen the excito-motor power of the spinal cord, by lessening the amount of blood circulating in it; and, second, to prevent those spasmodic contractions of the cerebral arteries which induce the sudden loss of consciousness constituting the first phase of an epileptic fit. To achieve these objects, I order—

*First*, and most important, ice to be applied to some one part or to the whole length of the back, and from two to eighteen hours a day, according to the special character of the case under treatment.

*Secondly*, if the extremities be cold, to aid them in recovering their wonted warmth during the first day or two of treatment—by frequently immersing them in hot water, and by friction, also in winter, by clothing the arms down to the wrists, and the legs down to the ankles, in flannel.

*Thirdly*, as auxiliaries, (1) to take abundant physical exercise, and to use dumb-bells when practicable, or other special means of increasing the respiratory activity and of expanding the energy of the spinal cord; (2) so to cut or dress the hair that it shall not cover or keep warm the upper part of the back of the neck; (3) to exercise the brain daily and systematically in some healthy study, or, if this be impracticable, to ensure regular mental activity by means of some interesting employment; and (4) to take care that the dress along the centre of the back be light and cool.

If ice be properly applied to the back, the extremities, however cold, may be made quickly warm, so that in many cases the use of hot water may be wholly dispensed with; but in severe cases, where immediate derivation of the blood to the extremities is urgently required, and more especially in winter, it is expedient to accelerate the influence of the ice applied to the sympathetic ganglia by the means just indicated.

The results of this method of treating epilepsy are exemplified in the following cases:—

*Case* 1.—A man, aged 42, began to have fits in 1854. During the twelve months previous to the beginning of my treatment (May 16) he had on an average, three fits of about twenty minutes' duration daily; since I began to treat him he has not had a single fit.

*Case* 2.—A girl, aged 17, began to have fits when between 13 and 14 years old. Has been accustomed since that time to have two little fits (*le petit mal*) daily. I began to treat her February 24, 1863. These little fits immediately became less pronounced, then having gradually lessened in number, ceased entirely at the end of the first week, and have never recurred.

*Case* 3.—A girl, aged 14, has had fits, chiefly little ones, for about six years. She becomes unconscious in each fit, but does not fall down. When I began to treat her, (April 23, 1863,) she was having fits at the rate of about four in each hour during the day, besides several each night. Each fit lasted from three to five minutes. The night fits ceased entirely in the middle of May, and the day fits, which now do not last above two or three seconds of time each fit, have declined at the following rates:

| | | | | |
|---|---|---|---|---|
| Total number of fits during | the week | ending | May 1, | 50 |
| " | " | " | " 8, | 65 |
| " | " | " | " 15, | 47 |
| " | " | " | " 22, | 37 |
| " | " | " | " 29, | 26 |
| " | " | " | June 5, | 11 |
| " | " | " | " 12, | 10 |
| " | " | " | " 19, | 8 |
| " | " | " | " 26, | 5 |
| " | " | " | July 3, | 6 |
| " | " | " | " 10, | 2 |

Note.—During the week ending May 8, the patient suffered much from toothache, and at length had two teeth drawn. Hence, I believe, the fact that she had more fits that week than the previous one.

*Case* 4.—A girl, aged 20, suffers from falling fits of the ordinary kind, lasting about three minutes each, from little fits, which she calls her "jerks," and in which she becomes unconscious, but does not fall down, and from a frequent quivering of the lips, which is a serious impediment to speech. Of the falling fits she usually has a large but uncounted number each month. In April last she had them continuously one after another throughout each day during a week. Of her little fits or "jerks," she usually had ten in each of the six days of her catamenial period, and one during each day of the interval. The quivering of the lips is a constant trouble. I began to treat her May 27; since that time she has had one falling fit, lasting about three minutes, and two lasting but an instant each; and since June 15, excepting one little "jerk," without losing consciousness, she has had no jerking fit, no quivering of the lips, and no abnormal symptom whatever.

*Case* 5.—A boy, aged 13, during the last twenty months has suffered from falling fits, and what his mother calls "stagnation fits," in which he becomes unconscious, but does not fall. On an average he had, until I began to treat him, about fifty of each kind of fit during each month. He came under my care June 4. Since that date he has had only one falling fit, which was induced by his brother, who made him angry, and has had no little fit whatever.

*Case* 6.—A boy, aged 14, in the habit of having an average of twelve fits daily, each preluded by a shriek. I began to treat him June 11. On that day he had four fits, but each of them without a shriek. Since that day he has not had a single fit.—*Medical Times and Gazette.*

